# Time-Course of Recovery Following a Strength–Power Emphasized Crossfit^®^ Microcycle

**DOI:** 10.3390/sports14070300

**Published:** 2026-07-14

**Authors:** Vinícius Emanoel Leal Pinto, Gisela Arsa, Manoella Regina de Souza Silva, Karine Naves de Oliveira Goulart, Julio Cerca Serrão, Jacielle Carolina Ferreira

**Affiliations:** 1 Research Center for Physical Education and Health, Federal University of Mato Grosso, Cuiabá 78060-900, Brazil; vini.emanoel.leal@gmail.com (V.E.L.P.); gisela.cunha@ufmt.br (G.A.); manucba@gmail.com (M.R.d.S.S.); karine.goulart@ufmt.br (K.N.d.O.G.); 2 School of Physical Education and Sport, University of São Paulo, São Paulo 05508-220, Brazil; jcserrao@usp.br

**Keywords:** countermovement jump, handgrip, total quality recovery, fatigue, readiness

## Abstract

Most studies investigating recovery in CrossFit^®^ have focused on the effects of a single training session, whereas the cumulative effects of consecutive sessions within a training microcycle remain poorly understood. This study aimed to evaluate perceived recovery and neuromuscular performance, assessed through the countermovement jump (CMJ) and handgrip strength tests, over a 72 h period following a habitual strength–power-emphasized CrossFit^®^ microcycle. Methods: Fourteen male CrossFit^®^ practitioners performed CMJ and handgrip strength tests and completed the Total Quality Recovery (TQR) scale before and at 24, 48, and 72 h after completing a strength–power-emphasized training microcycle. **Results:** Repeated-measures ANOVA revealed significant reductions in all variables 24 h after the microcycle compared with baseline values, followed by recovery to baseline within 48 h. **Conclusions:** These findings suggest that at least 48 h are required for the recovery of neuromuscular performance and perceived recovery following a habitual strength–power-emphasized CrossFit^®^ microcycle. Furthermore, the similar temporal responses observed across the three monitoring tools indicate that they may represent practical options for monitoring performance and recovery in CrossFit^®^ practitioners.

## 1. Introduction

CrossFit^®^ is a strength and conditioning modality characterized by constantly varied, functional, high-intensity movements designed to improve work capacity across multiple physical domains, including endurance, strength, and flexibility [1]. Training sessions typically incorporate gymnastic movements, Olympic weightlifting, cycling, running, and jump rope exercises. Owing to their high intensity and limited recovery intervals, these sessions may induce substantial levels of fatigue within a single training bout [2,3,4,5]. Compared with exercise protocols based on the guidelines of the American College of Sports Medicine (ACSM), Drum et al. [2] reported that CrossFit^®^ sessions elicited greater fatigue, muscle soreness, muscle swelling, shortness of breath, tenderness to light touch, and restricted movement of the exercised muscles. These findings suggest that CrossFit^®^ practitioners may experience greater post-exercise fatigue than individuals performing more conventional exercise protocols. Similarly, Santos et al. [6] reported higher maximal heart rate, blood lactate concentration, rating of perceived exertion (RPE), and perceived pain during CrossFit^®^ sessions than during high-intensity continuous training. Collectively, these findings indicate that CrossFit^®^ imposes distinct physiological demands compared with conventional exercise, highlighting the need for further investigation into the fatigue-recovery process associated with this training modality.

Several studies have examined the physiological and performance responses elicited by CrossFit^®^ training sessions. Some have compared different Workout of the Day (WOD) protocols, such as Barreto et al. [7], who compared Fran, Megan, and Diane, and Mota et al. [8], who compared Diane and Cindy. Others have investigated whether different repetition schemes within WODs produce distinct physiological or performance responses. For example, studies have compared the As Many Repetitions as Possible (AMRAP) and Round for Time (RFT) formats [4,5,9,10]. Overall, however, these investigations have primarily focused on the effects of a single CrossFit^®^ session, evaluating physiological and performance responses either during or immediately after exercise [5,6,7,8,9,10], or throughout recovery periods ranging from 24 to 72 h post-exercise [4,11,12,13]. In contrast, studies examining the cumulative effects of consecutive training sessions within a microcycle remain scarce [14].

In sports training, a subsequent training load is often applied before complete recovery from the previous session has occurred, meaning that athletes may begin a new training session while still experiencing residual fatigue. Within this framework, the supercompensation process is expected to occur following a sequence of training sessions rather than after an isolated session. This planned sequence of training sessions constitutes a microcycle, which represents one of the fundamental organizational units of training programming [15]. To the best of our knowledge, only one previous study has examined the effects of a CrossFit^®^ training microcycle on performance and perceived recovery [14]. Conde et al. [14] reported no reductions in countermovement jump (CMJ) performance or Total Quality Recovery (TQR) scores 24 h after strength- or aerobic-emphasized microcycles. However, important methodological aspects limit the interpretation and generalizability of those findings. The study included only seven female participants, assessed recovery at a single post-microcycle time point (24 h), and relied primarily on performance measures, with CMJ being the only assessment suitable for routine monitoring. In contrast, studies investigating isolated CrossFit^®^ sessions have consistently demonstrated fatigue-related responses 24 h after exercise. Rios et al. [11] reported reductions in strength performance relative to baseline, whereas Gomes et al. [13] observed elevated cortisol concentrations over the same period. Likewise, Timón et al. [4] found impaired strength performance accompanied by increased markers of muscle damage and blood glucose 24 h after two different Workout of the Day (WOD) protocols. Similarly, Sousa Neto et al. [12] reported reduced perceived recovery and elevated creatine kinase concentrations 24 h after the Karen WOD. Nevertheless, both Timón et al. [4] and Sousa Neto et al. [12] observed that these residual effects had returned to baseline values after 48 h, suggesting that this recovery period may be sufficient following a single CrossFit^®^ training session.

To better understand the fatigue-recovery state, practical and reliable monitoring tools are required. Although biochemical markers provide valuable physiological information, their routine application is often limited by cost and logistical constraints. Likewise, performance assessments such as specific CrossFit^®^ benchmark workouts, maximal oxygen uptake tests, and one-repetition maximum (1RM) tests may themselves induce additional fatigue while requiring substantial physical effort and participant motivation. Consequently, practitioners and researchers have increasingly adopted less demanding methods, including submaximal performance tests and subjective recovery scales, because of their practicality, low physical demand, and non-invasive nature. Among these tools, the countermovement jump (CMJ) is one of the most frequently used methods for monitoring neuromuscular performance and recovery across different sports, including CrossFit^®^ [3,4,10,12,14]. Another practical and low-cost alternative is handgrip strength assessment. Although no single measure fully reflects overall physical performance, handgrip strength has consistently been associated with several indicators of health and physical function. A recent systematic review demonstrated that approximately half of the available studies reported moderate to strong positive associations between handgrip strength and upper-limb, lower-limb, or trunk strength [16]. Furthermore, Cronin et al. [17] concluded that higher relative handgrip strength is advantageous in sports requiring sustained grip force, body-weight support, or repeated interaction with external implements. The same review also reported strong correlations between handgrip strength and powerlifting performance (i.e., bench press, squat, and deadlift), particularly among raw powerlifters, suggesting that handgrip strength reflects overall neuromuscular performance across different strength-related tasks. Considering that CrossFit^®^ combines weightlifting exercises with gymnastic movements that impose substantial demands on grip strength, handgrip assessment may represent a useful complementary tool for monitoring neuromuscular performance and the fatigue-recovery state.

Although CMJ and handgrip strength assessments are widely used as performance measures, they were not originally developed to quantify recovery status. In contrast, the Total Quality Recovery (TQR) scale was specifically designed to assess psychophysiological recovery and is structured similarly to the Borg 6–20 Rating of Perceived Exertion scale [18]. Evidence supporting its applicability includes the strong negative association between TQR scores and creatine kinase concentrations following professional soccer matches [19]. In CrossFit^®^, Sousa Neto et al. [12] also demonstrated reduced TQR scores 24 h after the Karen WOD, with values returning to baseline after 72 h. These findings suggest that subjective recovery measures may be sensitive to changes in the fatigue-recovery state. Moreover, combining subjective and objective measures may provide a more comprehensive assessment of athletes’ recovery status.

Despite the growing popularity of CrossFit^®^, the recovery time course following habitual training microcycles remains poorly understood. Considering that complete recovery between consecutive training sessions is not always achieved, particularly among practitioners with high weekly training frequency, further investigation into the cumulative effects of training microcycles is warranted. Therefore, the present study aimed to evaluate perceived recovery and neuromuscular performance, assessed using the countermovement jump (CMJ), handgrip strength, and the Total Quality Recovery (TQR) scale, over a 72 h period following a habitual strength–power-emphasized CrossFit^®^ microcycle. By extending the monitoring period beyond previous investigations and combining practical objective and subjective assessment tools, this study provides novel information regarding the temporal pattern of performance and recovery following habitual CrossFit^®^ training. We hypothesized that the investigated microcycle would induce transient impairments in neuromuscular performance and perceived recovery, with the greatest reductions occurring 24 h after the microcycle, followed by progressive recovery over the subsequent 72 h.

## 2. Materials and Methods

### 2.1. Sample and Ethical Considerations

This study was conducted with male CrossFit^®^ practitioners who trained regularly. The sample size was calculated based on data reported by Oliver-López et al. [10], using countermovement jump (CMJ) performance and a paired-samples t-test, assuming a significance level of 0.05, an effect size (Cohen’s d) of 0.80, and a statistical power of 80%. The sample size calculation indicated that a minimum of 12 participants was required. To account for potential attrition, 25 participants were initially recruited. Of these, 11 participants missed at least one training session or testing session and were therefore excluded from the study. Consequently, 14 participants completed all experimental procedures (mean ± SD: age, 28.93 ± 4.75 years; body mass, 83.36 ± 9.64 kg; height, 177.07 ± 6.43 cm; training experience, 3.43 ± 1.55 years).

The inclusion criteria were as follows: (1) male participants aged between 18 and 40 years; (2) current registration and active participation at a CrossFit^®^ affiliate; (3) a minimum of eight months of CrossFit^®^ experience; and (4) no diagnosis of chronic or osteoarticular diseases, bone fractures, or joint prostheses. Participants who failed to complete any stage of the study were excluded from the analysis. The study protocol was approved by the local Research Ethics Committee, and all participants provided written informed consent prior to participation.

### 2.2. Experimental Design

**Figure 1:**
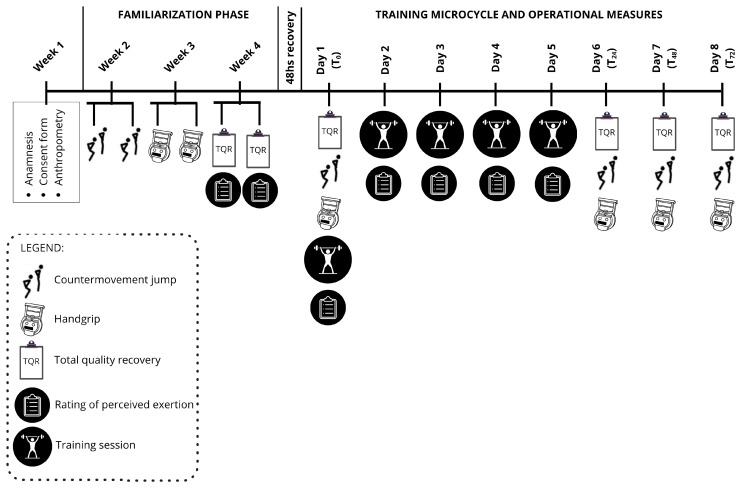
Experimental design of the study. Figure created with Canva (https://www.canva.cn/en/?display-com-option=true (accessed on 1 July 2026)).

The study consisted of an initial session for medical history assessment (anamnesis), anthropometric measurements, and the provision of written informed consent, followed by a familiarization phase with the experimental procedures (performance tests, perceived recovery, and perceived exertion). Subsequently, participants completed the experimental training phase, during which the operational measures were collected before and after the training microcycle. The microcycle consisted of five consecutive days of CrossFit^®^ training sessions. The operational measures used to assess the fatigue-recovery state included the countermovement jump (CMJ), handgrip strength, and the Total Quality Recovery (TQR) scale, which were assessed at baseline and at 24, 48, and 72 h after completion of the microcycle (Figure 1).

### 2.3. Familiarization Phase

Over a three-week period, two familiarization sessions were conducted per week, with a 48 h interval between sessions. In the first week, participants performed three CMJ attempts before beginning training. In the second week, the same procedure was carried out using the handgrip test. In the third week, participants were familiarized with the TQR scale by indicating the value corresponding to their perceived level of recovery prior to the start of training. Participants then performed the CrossFit^®^ training session planned for the day, and, following the session, one of the researchers administered the RPE scale, which, during the main phase, was used to quantify the load of each training session.

### 2.4. Training and Operational Measures Phase

During the experimental phase, participants completed a habitual strength-pow-emphasized CrossFit^®^ microcycle, consisting of five consecutive training sessions separated by 24 h intervals (Appendix A). The microcycle was designed by the head coach of the affiliated CrossFit^®^ facility, and the researchers had no involvement in planning or modifying the training program.

The microcycle was classified as strength–power-emphasized because its primary training objective was to develop maximal strength and power through resistance and weightlifting exercises. Each training session included dedicated strength and/or Olympic weightlifting components (e.g., back squat, deadlift, clean, snatch, and their derivatives), which were generally performed before the conditioning segment, with progressive loading when appropriate. Although all sessions also included metabolic conditioning (AMRAP or “for time” workouts), these conditioning components predominantly incorporated weightlifting and other strength-related movements. Therefore, the overall emphasis of the microcycle was considered to be the development of strength and power rather than aerobic or metabolic conditioning alone.

All participants completed the same five training sessions, following an identical exercise sequence and workout structure. External loads were individually prescribed according to each participant’s training level and the coach’s programming, consistent with the routine practices of the affiliated CrossFit^®^ facility. Participants were instructed to refrain from any additional physical training during the 48 h preceding the beginning of the microcycle and throughout the subsequent 72 h recovery period after its completion. During this recovery period, participants attended the laboratory only for the scheduled monitoring assessments.

On the first day of the microcycle, prior to the training session, participants performed the operational tests that served as baseline measures (T0), using the same procedures during the familiarization sessions. Upon arriving at the training facility, the volunteers completed the TQR, performed the CMJ, and subsequently the handgrip test. The operational tests were repeated at three additional time points: 24 h after the last training session (T24), 48 h after the last training session (T48), and 72 h after the last training session (T72). After each session of the microcycle, participants reported their rating of perceived exertion (RPE) to one of the researchers, who also recorded the duration of the training sessions to characterize the training load during the microcycle. Data collection was conducted at the CrossFit^®^ gym where the participants regularly trained. In both the familiarization and main phases, the procedures for motor tests and recovery assessment followed the protocols described below.

Countermovement Jump (CMJ): The CMJ familiarization protocol consisted of a warm-up comprising three sets of 10 jumping jacks, 5 jump squats, and 10 alternating high-knee raises (stationary running). Upon completion of the warm-up, the volunteers performed three jumps with a one-minute interval between attempts. For the execution of the CMJ, participants started from an upright standing position, with hands on the hips, feet parallel and approximately shoulder-width apart. They then flexed the hips, knees, and ankles, followed by a maximal vertical jump, maintaining their hands on the hips throughout the movement. The highest jump achieved across the three attempts was retained for subsequent analysis. Familiarization and jump testing were conducted using a contact mat (MultiSprint—Hidrofit^®^, Belo Horizonte, Brazil), which estimates jump height based on flight time [20].

Handgrip Strength: Handgrip strength was assessed bilaterally using a load cell-instrumented dynamometer connected to an HX711 amplifier and an Arduino UNO (Arduino^®^, Monza, Italy) platform [21]. The dynamometer was calibrated before data collection using known masses ranging from 3 to 70 kg. Calibration accuracy was subsequently verified before each testing session using a 25 kg reference mass, yielding consistent measurements throughout the study. Participants completed a warm-up consisting of three submaximal handgrip contractions of five seconds each, with 15 s intervals between efforts, followed by a three-second maximal voluntary isometric contraction (MVIC) on the dynamometer. One minute after the warm-up, the maximal handgrip strength test was conducted. The assessment consisted of one 10 s maximal contraction with the right hand, followed by a one-minute recovery interval and a subsequent 10 s maximal contraction with the left hand. The testing order was standardized for all participants, regardless of hand dominance, to ensure consistency across assessments. The test was performed with the participant seated on a chair with back support and no armrests, and the elbow flexed at a 90° angle. Force data were sampled at 80 Hz, exported in .txt format, and processed using Matlab^®^ v. 2017a software to determine the maximal force value, defined as the highest force recorded during each trial.

Total Quality Recovery Scale (TQR): The Total Quality Recovery (TQR) scale was used to assess perceived recovery, providing a practical measure of the participants’ psychophysiological recovery status [18]. During each assessment, participants were asked the following question: “How do you feel regarding your recovery?” They then selected the score that best represented their perceived recovery on a scale ranging from 6 (“not recovered at all”) to 20 (“fully recovered”). The TQR scale was administered immediately before each assessment session.

Rating of Perceived Exertion Scale (RPE): The Rating of Perceived Exertion (RPE) scale proposed by Borg [22] was used to quantify the internal training load of each training session. Following each training session, participants were asked “How was your training session?” They then reported a score from 1 to 10 that best represented their perceived exertion. To minimize the influence of the final exercise performed during the training session, RPE was collected between 15 and 30 min after exercise completion. Internal training load was calculated according to the method proposed by Foster et al. [23] by multiplying the session RPE by the session duration (min), with the result expressed in arbitrary units (a.u.).

### 2.5. Statistical Analyses

**Figure 2:**
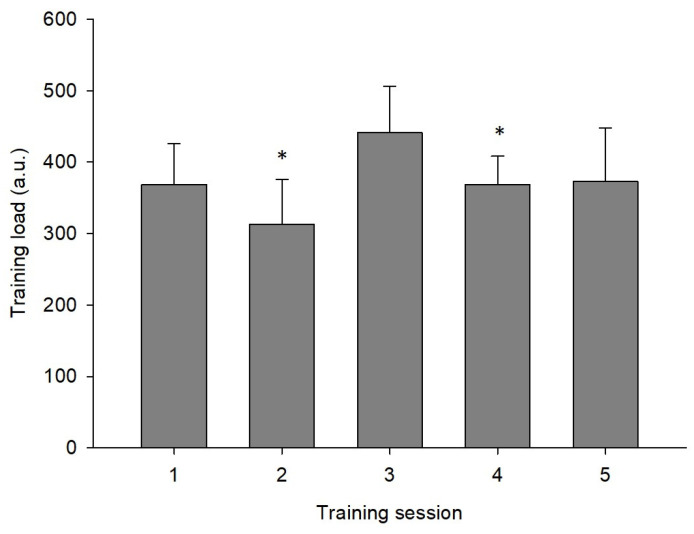
Mean and standard deviation of strength–power-emphasized microcycle training load. Legend: a.u. = arbitrary units; * Significant difference compared with session 3.

**Figure 3:**
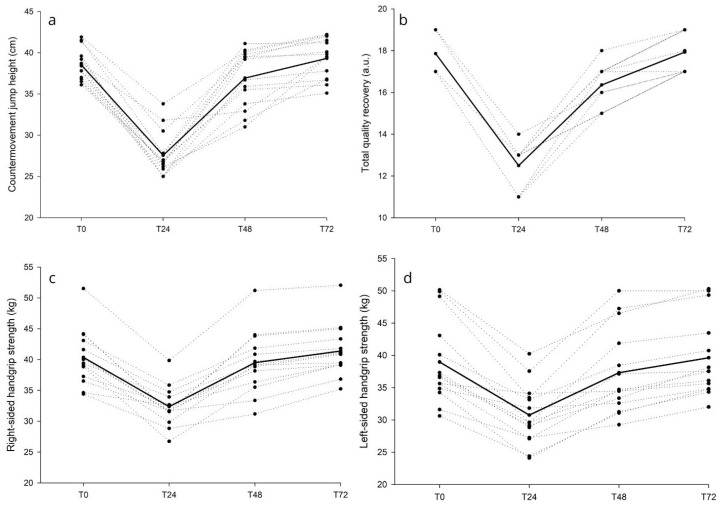
Scatter plot of operational measures results following the strength–power-emphasized microcycle. (**a**) Countermovement jump height; (**b**) Total quality recovery; (**c**) right-hand handgrip strength; (**d**) left-hand handgrip strength; T0 = Baseline; T24 = 24 h post-microcycle; T48 = 48 h post-microcycle; T72 = 72 h post-microcycle; dashed black line = individual results; thick solid line = average.

Statistical analyses and graphical representations of the results (Figure 2 and Figure 3) were performed using Jamovi (https://www.jamovi.org/index.html (accessed on 1 July 2026)) and Sigma Plot 10.0. Data normality was assessed using the Shapiro–Wilk test, and sphericity was assessed using Mauchly’s test. When the assumption of sphericity was violated, the Greenhouse–Geisser correction was applied. Differences in the dependent variables (CMJ, Handgrip, and TQR score) across baseline (T0) and 24 (T24), 48 (T48), and 72 h (T72) post-microcycle were analyzed using a one-way repeated-measures ANOVA, followed by Tukey’s post hoc test when appropriate. Effect sizes for ANOVA were reported as eta squared (η^2^), and post hoc effect sizes were calculated using Cohen’s d, defined as the mean difference between time points divided by the pooled standard deviation. The same statistical procedures and assumption checks were applied to the training load data. Differences in training load across the five sessions were also analyzed using a one-way repeated-measure ANOVA. Data are presented as mean ± standard deviation. Statistical significance was set at *p* < 0.05.

## 3. Results

The training load of the microcycle was quantified using the method proposed by Foster et al. [23], and the descriptive results are presented in Figure 2. The ANOVA revealed a significant effect (*p* < 0.01; F_(3, 52)_ = 6.85; η^2^ = 0.32). Post hoc analysis showed that session 3 exhibited a higher training load compared with sessions 2 (*p* = 0.01; d = 2.01) and 4 (*p* = 0.04; d = 1.35), whereas no significant difference was observed between sessions 3 and session 1 (*p* = 0.08; d = 1.19) or session 5 (*p* = 0.15; d = 0.98). No other comparisons showed significant differences (*p* > 0.05). The total training load of the microcycle was 1864.29 ± 89.50 arbitrary units (a.u.), and the mean RPE across all sessions was 6.21 (corresponding to “strong” to “very strong”).

Regarding the operational measures, CMJ performance was significantly reduced at T24 compared with T0 (*p* = 0.01; d = 4.66), indicating impaired performance 24 h after the microcycle. Performance at T24 was also significantly lower than at T48 (*p* = 0.01; d = 3.03) and T72 (*p* = 0.01; d = 4.61). Furthermore, performance at T48 remained significantly lower than at T72 (*p* = 0.02; d = 0.79). No significant differences were observed between T0 and T48 (*p* = 0.34; d = 0.56), or between T0 and T72 (*p* = 0.44; d = 0.36).

Similarly, handgrip strength decreased at T24 for both hands compared with T0 (right: *p* = 0.01; d = 2.06; left: *p* = 0.01; d = 1.44). Performance at T24 was also significantly lower than at T48 (right: *p* = 0.01; d = 1.72; left: *p* = 0.01; d = 1.15) and T72 (right: *p* = 0.01; d = 2.45; left: *p* = 0.01; d = 1.61). For the right hand, performance at T72 was significantly greater than at T0 (*p* = 0.02; d = 0.24), whereas for the left hand, performance at T48 remained significantly lower than at T0 (*p* = 0.01; d = 0.25).

TQR scores were significantly lower at T24 (*p* = 0.01; d = 5.23) and T48 (*p* = 0.01; d = 1.47) compared with T0. Additionally, TQR scores were significantly higher at T48 (*p* = 0.01; d = 3.80) and T72 (*p* = 0.01; d = 5.38) compared with T24. Moreover, TQR scores at T72 were significantly higher than at T48 (*p* = 0.01; d = 1.56), indicating a progressive increase in perceived recovery over time. Table 1 presents the CMJ, handgrip, and TQR values at baseline and post-microcycle time points. Figure 3 (Graphs A-D) illustrates the individual responses for each of the measured variables.

## 4. Discussion

This study aimed to assess perceived recovery and neuromuscular performance, using CMJ and handgrip strength tests, over a 72 h period following a habitual CrossFit^®^ microcycle predominantly focused on strength–power development. The results indicate that this type of microcycle induces a significant reduction in performance 24 h after its completion, with most performance measures recovering to baseline within 48 h, except for left-hand handgrip strength. In the context of sports training, residual training effects may manifest in different directions: positive effects, associated with performance enhancement (e.g., increased protein synthesis), and negative effects, related to performance decrements (e.g., neuromuscular fatigue) [24]. As training adaptation is characterized by a dynamic interaction between training stress and recovery [25], temporary reductions in performance are expected following planned training sessions or microcycles. Therefore, systematic monitoring of training responses is essential to guide decision-making, optimize training load management, and maintain an appropriate balance between training stress and recovery [24,26,27]. In the present sample, performance-related measures generally returned to values comparable to baseline approximately 48 h after completion of the investigated strength–power-focused CrossFit^®^ microcycle.

Previous studies have used the CMJ as a tool to monitor performance and recovery in CrossFit^®^. Martínez-Gómez et al. [28] investigated different laboratory tests as predictors of performance in this modality and observed that, although performance is multifactorial, CMJ performance combined with maximal oxygen consumption explained a large proportion of the variance in overall performance (R^2^ = 0.81). Given this finding and the widespread use of CMJ as a functional test, it has been applied to identify post-exercise performance decrements in CrossFit^®^. However, the available evidence is inconsistent. Rios et al. [11] observed a reduction in jump performance 24 h after performing the “Fran” WOD. In contrast, Sousa Neto et al. [12] reported an immediate post-exercise decrease, with a return to baseline values after 24 h in a protocol based on the “Karen” WOD. Timón et al. [4] reported no changes in CMJ performance 24 or 48 h after different training schemes (AMRAP vs. RFT). These discrepancies may be related to variability in training session characteristics across studies, including volume, intensity, exercise selection, and stimulus organization. Consequently, it remains challenging to establish CMJ as a consistent tool for monitoring performance and recovery status following isolated sessions or between training sessions in CrossFit^®^.

Following accumulated training sessions, information on the time course of recovery and applicable monitoring tools in CrossFit^®^ remains limited. To date, only one study has investigated the effects of a strength-emphasized microcycle on CMJ performance, reporting findings that differ from those of the present study. Conde et al. [14] applied a five-session microcycle composed of gymnastic strength exercises (bar work, handstands, single-leg squats, etc.) and short, high-intensity strength training bouts, and did not find significant changes in CMJ performance between pre-microcycle assessment and 24 h after the last training session. Although both studies employed five-session microcycles, methodological differences may help explain these contrasting findings. In the study by Conde et al. [14], the sample consisted of only seven women, whereas the present study included a larger sample of male participants. In addition, the small sample size, combined with high data variability (coefficient of variation of approximately 29% in the pre-microcycle assessment: 19.43 ± 5.75 cm), may have limited statistical power to detect significant differences. Even though these factors may partially account for the discrepancies between studies, such interpretations remain speculative, as they were not directly investigated in either study. Nevertheless, both studies are among the first to examine the recovery time course in CrossFit^®^ following training microcycles. Therefore, the results should be interpreted with caution, particularly regarding the use of CMJ as a tool for monitoring residual training effects, highlighting the need for further investigation in more ecologically valid contexts, such as real training microcycles.

In contrast to CMJ, handgrip strength remains underexplored as a tool for monitoring training responses in healthy individuals. Previous studies have primarily examined its ability to detect localized fatigue [29,30,31], whereas evidence regarding its responsiveness to whole-body training stress remains limited. Nevertheless, handgrip strength has also been proposed as an important biomarker of overall health, given its association with outcomes such as mortality, morbidity, hospitalization, physical function, and psychological health [32]. Although these associations are primarily based on chronic health outcomes rather than acute training responses, they suggest that handgrip strength may reflect both local muscular demands and broader physiological responses; however, its role in monitoring training responses has not yet been clearly established. In the present study, handgrip performance followed a temporal pattern similar to that observed for CMJ and TQR, decreasing 24 h after the microcycle and generally returning to baseline values after 48 h. These findings do not establish handgrip strength as a valid tool for monitoring short-term training responses; however, they indicate that its potential in this context warrants further investigation, particularly following training microcycles characterized by higher load accumulation. One possible explanation for these findings is that CrossFit^®^ training combines substantial whole-body demands with exercises requiring sustained grip force. Gymnastic movements such as pull-ups, as well as weightlifting exercises, require continuous force production by the forearm muscles to support body weight or maintain control of the barbell. According to Cronin et al. [17], stronger associations between handgrip strength and sport performance tend to be observed in activities requiring sustained force application to implements or continuous support of body weight. Although this mechanism cannot fully explain the present findings, it suggests that the reduction in handgrip performance observed after the microcycle may reflect, at least in part, the high local demands imposed on the forearm musculature, together with the overall physiological stress induced by training.

Among the monitoring tools evaluated in the present study, the TQR scale was the only measure providing an internal assessment independent of physical performance. Developed by Kenttä and Hassmén [18], the TQR scale aims to quantify psychophysiological recovery and is structured analogously to the rating of perceived exertion (RPE) scale, facilitating its practical application in sports settings. In the present study, TQR showed a significant decrease 24 h after the microcycle, followed by a progressive increase at subsequent time points, with values at 48 h higher than at 24 h and at 72 h higher than at 48 h. Moreover, among all evaluated tools, TQR exhibited the largest effect size, suggesting a greater magnitude of change across the recovery period compared with the other measures. Similar results were reported by Sousa Neto et al. [12], who observed a reduction in TQR 24 h after a “Karen” WOD session, followed by recovery at 72 h. Conversely, Conde et al. [14] did not find changes in TQR 24 h after a strength-emphasized microcycle. Methodological differences between studies, as previously discussed, may contribute to these divergent findings. Nevertheless, TQR has been shown to be a viable tool for monitoring recovery processes across different sports modalities, including basketball [33], volleyball [34], and soccer [19]. Collectively, these findings reinforce the potential of TQR as a practical tool for monitoring perceived recovery status.

The results of the present study have important practical implications. First, the findings from CMJ, handgrip strength, and TQR consistently suggest that experienced male practitioners may require at least 48 h of recovery to restore neuromuscular performance and perceived recovery following the strength–power-emphasized microcycle investigated in the present study. Second, the three tools used to assess performance and recovery status showed similar temporal responses following the applied microcycle. This finding supports the feasibility of using low-cost, easy-to-administer methods with minimal physical and time demands, facilitating their adoption by coaches in practical settings. However, some limitations should be considered. The absence of objective physiological markers, such as creatine kinase (CK), limits a more comprehensive interpretation of participants’ internal state. Although no single marker is considered a gold standard, the inclusion of physiological measures could complement the assessment of fatigue and recovery status. Regarding procedures, participants were instructed to refrain from physical training before and during the testing period; however, sleep, hydration status, and nutritional intake were not controlled. These are well-established modulators of post-exercise recovery, influencing neuromuscular function, metabolic restoration, and perceptual responses [35,36,37]. These uncontrolled variables may have contributed to the inter-individual variability observed in recovery responses. Therefore, part of the dispersion in the dependent variables across time points may be explained by differences in these external and behavioral factors, which were not monitored in the present study. In addition, the sample size was small and consisted exclusively of male participants. Given that previous studies involving female participants have reported different results, such as Conde et al. [14], sex-related differences in fatigue and recovery responses may exist. Finally, because the response pattern of the dependent variables was observed following a specific strength–power-emphasized CrossFit^®^ microcycle, the findings should not be generalized to other training loads or microcycle designs. Moreover, no control group or alternative condition was included for comparison. Therefore, future studies should include additional physiological markers and compare fatigue and recovery responses between male and female participants following CrossFit^®^ microcycles.

## 5. Conclusions

Based on the present findings, at least 48 h of recovery are required for the restoration of neuromuscular performance (CMJ and handgrip strength) and perceived recovery to baseline levels following a habitual strength–power-emphasized CrossFit^®^ microcycle in male practitioners. Furthermore, the similar temporal patterns observed across the three monitoring tools suggest that they may represent practical options for monitoring performance and recovery status in CrossFit^®^ practitioners.

## Figures and Tables

**Table 1:** Results of operational tests performed before and after 24, 48, and 72 h post-training microcycle.

Variable	T0	T24	T48	T72	ANOVA		
	Mean ± SD (CI)	Mean ± SD (CI)	Mean ± SD (CI)	Mean ± SD (CI)	*p*	F	ES (η^2^)
CMJ (cm)	38.51 ± 1.97 (37.4–39.7)	27.56 ± 2.67 ^a^ (26.0–29.1)	36.69 ± 3.97 ^b^ (34.9–38.9)	39.31 ± 2.42 ^b,c^ (37.9–40.7)	0.01	F_(3, 39)_ = 103	0.77
Handgrip-R (kg)	40.35 ± 1.19 (37.8–42.9)	32.37 ± 0.84 ^a^ (30.5–34.2)	39.51 ± 1.32 ^b^ (36.7–42.4)	41.39 ± 1.10 ^a,b,c^ (39.0–43.8)	0.01	F_(1.4, 18.5)_ = 75.8	0.43
Handgrip-L (kg)	38.98 ± 1.76 (35.2–42.8)	30.75 ± 1.24 ^a^ (28.1–33.4)	37.38 ± 1.76 ^a,b^ (33.5–41.1)	39.63 ± 1.66 ^b,c^ (36.0–43.2)	0.01	F_(1.25, 16.3)_ = 52.5	0.27
TQR (a.u.)	17.86 ± 1.03 (17.3–18.5)	12.50 ± 1.02 ^a^ (11.9–13.1)	16.36 ± 1.01 ^a,b^ (15.8–16.9)	17.93 ± 1.00 ^b,c^ (17.4–18.5)	0.01	F_(1.8, 23.43)_ = 126	0.84

T0 = Baseline; T24 = 24 h post-microcycle; T48 = 48 h post-microcycle; T72 = 72 h post-microcycle; SD = Standard deviation; CI = Confidence interval; ES = Effect size; η^2^ = Squared Eta; CMJ = Countermovement jump; R = Right hand; L = Left hand; TQR = Total quality recovery; ^a^ Significant difference compared to T0; ^b^ Significant difference compared to T24; ^c^ Significant difference compared to T48.

## Data Availability

The data presented in this study are available on reasonable request from the corresponding author.

## Abbreviations

The following abbreviations are used in this manuscript:

ACSM	American College of Sports Medicine
AMRAP	As many repetitions as possible
CMJ	Countermovement jump
CI	Confidence interval
ES	Effect size
MVIC	Maximal voluntary isometric contraction
η^2^	Eta squared
RPE	Rating of perceived exertion
RFT	Round for time
SD	Standard deviation
T24	24 h after the last training session
T48	48 h after the last training session
T72	72 h after the last training session
TQR	Total Quality Recovery
WOD	Workout of the Day

## Appendix A

**Table A1:** Strength–power-oriented microcycle.

	Training 1	Training 2	Training 3	Training 4	Training 5
Part 1	5 min Mobility Tabata 20 s on 10 s off Seated leg Lift over Kettlebell Cossak Squat	4 min Mobility 30″ on 30″ off Snatch balance Hang Power Snatch (40/25)	5 min Mobility Tabata Archie rock Hollow rock	5 min Mobility AMRAP 10 Stiff 10 Abdominal Leg raise 10 Lunge	5 min Mobility 3 Rounds 20 Jump Jacks 4 Muscle Clean (50/35) 8 Sprawl
Part 2	10 min 5 rounds (biset) 6 Clean High Pull (70/45) 20 Weighted full sit-up	12 min 10 rounds Power Snatch Hang Snatch * Increase the load if possible	15 min Back Squat 10–8–6–4–2 * Load progression	10 min 5 rounds 15 Dual Dumbbell row 8 Deadlift (100/65)	Explanation and preparation for the day’s workout
Part 3	AMRAP 15′ 2–4–6–8... Clean (50/40) 10 Step over box Kettlebell 20 Kettlebell swing	For Time 14′ 21–15–9 Thruster Snatch (50/35) Time remaining: 2 reps max of thruster	AMRAP 14′ 200 m Running 20 Sit up 20 Box Jump Over	FT 15′ 5 rounds 20 Pull up 20 Wall ball 20 Dumbbell Snatch	AMRAP 8′ Buy in:100 Double Under (2×) 4 Hang Cleans (50/35) 8 Burpees target rest 2′ AMRAP 8′ Buy in: 100 Double Under 4 Power clean (60/45) 8 Burpee Over the Bar rest 2′ For Time: 10′ 3 rounds 80 Double Under 4 Power clean (80/50)

## References

[1] Glassman G. (2002). What is fitness. CrossFit J..

[2] Drum S.N., Bellovary B.N., Jensen R.L., Moore M.T., Donath L. (2017). Perceived demands and postexercise physical dysfunction in CrossFit^®^ compared to an ACSM based training session. J. Sports Med. Phys. Fit..

[3] Matea-Muñoz J.L., Lougedo J.H., Barba M., García-Fernández P., Garnacho-Castaño M.V., Domínguez R. (2018). Muscular fatigue in response to different modalities of CrossFit sessions. PLoS ONE.

[4] Timón R., Olcina G., Camacho-Cardeñosa M., Camacho-Cardenosa A., Martinez-Guardado I., Marcos-Serrano M. (2019). 48-hour recovery of biochemical parameters and physical performance after two modalities of CrossFit workouts. Biol. Sport.

[5] Toledo R., Dias M.R., Toledo R., Erotides R., Pinto D.S., Reis V.M., Novaes J.S., Vianna J.M., Heinrich K.M. (2021). Comparison of physiological responses and training load between different CrossFit^®^ workouts with equalized volume in men and women. Life.

[6] Santos D.A.T., Morais N.S., Viana R.B., Costa G.C.T., Andrade M.S., Vancini R.L., Weiss K., Knechtle B., de Lira C.A.B. (2025). Comparison of physiological and psychobiological acute responses between high intensity functional training and high intensity continuous training. Sports Med. Health Sci..

[7] Barreto A.C., Medeiros A.P., da Silva Araujo G., Vale R., Vianna J.M., Alkimin R., Serra R., Leitão L., Reis V.M., da Silva Novaes J. (2023). Heart rate variability and blood pressure during and after three CrossFit^®^ sessions. Retos.

[8] Mota M.R., Brandão H.C.P., Alencastro G., Elias R., Ribeiro A., de Araujo Ribeiro A.L., Chaves S.N., Cleto F., Silva A.O., Clael S. (2021). Glycemia analysis in two different CrossFit^®^ benchmark protocols. J. Exerc. Physiol. Online.

[9] Forte L.D.M., Freire Y.G.C., Júnior J.S.D.S., Melo D.A., Meireles C.L.S. (2022). Physiological responses after two different CrossFit workouts. Biol. Sport.

[10] Oliver-López A., García-Valverde A., Sabido R. (2025). Acute effect of three functional fitness training designs with equalized load on inexperienced and experienced athletes. PeerJ.

[11] Rios M., Becker K.M., Monteiro A.S., Fonseca P., Pyne D.B., Reis V.M., Moreira-Gonçalves D., Fernandes R.J. (2024). Effect of the Fran CrossFit workout on oxygen uptake kinetics, energetics, and postexercise muscle function in trained CrossFitters. Int. J. Sports Physiol. Perform..

[12] De Sousa Neto I.V., de Sousa N.M.F., Neto F.R., Neto J.H.F., Tibana R.A. (2022). Time course of recovery following CrossFit^®^ Karen benchmark workout in trained men. Front. Physiol..

[13] Gomes J.H., Mendes R.R., Franca C.S., Da Silva-Grigoletto M.E., Pereira da Silva D.R., Antoniolli A.R., de Oliveira e Silva A.M., Quintans-Júnior L.J. (2020). Acute leucocyte, muscle damage, and stress marker responses to high-intensity functional training. PLoS ONE.

[14] Conde T.F., Silva M.R.S., Caobianco J., Robalino J., Ferreira J.C. (2022). Sensitivity of operational tests to training load in CrossFit^®^. J. Phys. Educ. Sport.

[15] Matveyev L.P. (1981). Fundamentals of Sports Training.

[16] Szaflik P., Zadoń H., Michnik R., Nowakowska-Lipiec K. (2025). Handgrip strength as an indicator of overall strength and functional performance: Systematic review. Appl. Sci..

[17] Cronin J., Lawton T., Harris N., Kilding A., McMaster D.T. (2017). A brief review of handgrip strength and sport performance. J. Strength Cond. Res..

[18] Kenttä G., Hassmén P. (1998). Overtraining and recovery: A conceptual model. Sports Med..

[19] Osiecki R., Rubio T.B.G., Coelho R.L., Novack L.F., Conde J.H.S., Alves C.G., Malfatti C.R.M. (2015). The total quality recovery scale (TQR) as a proxy for determining athletes’ recovery state after a professional soccer match. J. Exerc. Physiol. Online.

[20] Ferreira J.C., Carvalho R.G.D.S., Szmuchrowski L.A. (2008). Validade e confiabilidade de um tapete de contato para mensuração da altura do salto vertical. Rev. Bras. Biomec..

[21] Cavalcante A., Robalino J.A., Santos P., Costa V.C., Ferreira J.C. (2023). Validade e confiabilidade da força de preensão manual avaliada com um dinamômetro de baixo custo. Rev. Remecs-Rev. Multidiscip. Estud. Científicos Saúde.

[22] Borg G.A. (1982). Psychophysical bases of perceived exertion. Med. Sci. Sports Exerc..

[23] Foster C., Florhaug J.A., Franklin J., Gottschall L., Hrovatin L.A., Parker S., Doleshal P., Dodge C. (2001). A new approach to monitoring exercise training. J. Strength Cond. Res..

[24] Jeffries A.C., Marcora S.M., Coutts A.J., Wallace L., McCall A., Impellizzeri F.M. (2022). Development of a revised conceptual framework of physical training for use in research and practice. Sports Med..

[25] Issurin V.B. (2010). New horizons for the methodology and physiology of training periodization. Sports Med..

[26] Meeusen R., Duclos M., Foster C., Fry A., Gleeson M., Nieman D., Raglin J., Rietjens G., Steinacker J., Urhausen A. (2013). Prevention, diagnosis, and treatment of the overtraining syndrome: Joint consensus statement of the European College of Sport Science and the American College of Sports Medicine. Med. Sci. Sports Exerc..

[27] Halson S.L. (2014). Monitoring training load to understand fatigue in athletes. Sports Med..

[28] Martínez-Gómez R., Valenzuela P.L., Alejo L.B., Gil-Cabrera J., Montalvo-Pérez A., Talavera E., Lucia A., Moral-González S., Barranco-Gil D. (2020). Physiological predictors of competition performance in CrossFit athletes. Int. J. Environ. Res. Public Health.

[29] Souza V.K., Claudino A.F., Kuriki H.U., Marcolino A.M., Fonseca M.d.C.R., Barbosa R.I. (2017). Fatigue of the wrist extensor muscles decreases palmar grip strength. Fisioter. Pesqui..

[30] Ohtaka C., Yanagita K., Nakata H., Fujiwara M., Shibasaki M. (2025). Effects of muscular fatigue on the performance of handgrip tasks during force generation and relaxation. J. Mot. Behav..

[31] Forman G.N., Sonne M.W., Kociolek A.M., Gabriel D.A., Holmes M.W.R. (2022). Influence of muscle fatigue on motor task performance of the hand and wrist: A systematic review. Hum. Mov. Sci..

[32] Vaishya R., Misra A., Vaish A., Ursino N., D’ambrosi R. (2024). Hand grip strength as a proposed new vital sign of health: A narrative review of evidences. J. Health Popul. Nutr..

[33] Sansone P., Tschan H., Foster C., Tessitore A. (2020). Monitoring training load and perceived recovery in female basketball: Implications for training design. J. Strength Cond. Res..

[34] Miranda-Mendoza J., Hernández-Cruz G., Reynoso-Sánchez L.F., González-Fimbres R.A., Cejas-Hernández B.A. (2023). Control of recovery using the Total Quality Recovery (TQR) scale during four accumulation microcycles and its relationship to physiological factors. Retos.

[35] Fullagar H.H.K., Skorski S., Duffield R., Hammes D., Coutts A.J., Meyer T. (2015). Sleep and athletic performance: The effects of sleep loss on exercise performance, and physiological and cognitive responses to exercise. Sports Med..

[36] Thomas D.T., Erdman K.A., Burke L.M. (2016). Position of the Academy of Nutrition and Dietetics, Dietitians of Canada, and the American College of Sports Medicine: Nutrition and athletic performance. J. Acad. Nutr. Diet..

[37] Sawka M.N., Burke L.M., Eichner E.R., Maughan R.J., Montain S.J., Stachenfeld N.S. (2007). Exercise and fluid replacement. Med. Sci. Sports Exerc..

